# Control of *Escherichia coli* in Poultry Using the In Ovo Injection Technique

**DOI:** 10.3390/antibiotics13030205

**Published:** 2024-02-22

**Authors:** Gabriel da Silva Oliveira, Concepta McManus, Vinícius Machado dos Santos

**Affiliations:** 1 Faculty of Agronomy and Veterinary Medicine, University of Brasília, Brasília 70910-900, Brazil; gabriels.unb@gmail.com (G.d.S.O.);; 2 Laboratory of Poultry Science, Federal Institute of Brasília—Campus Planaltina, Brasília 73380-900, Brazil

**Keywords:** antimicrobials, egg microbiology, in-ovo injection, microbial reduction, poultry microbiology, poultry safety

## Abstract

Pathogens, such as *Escherichia coli* (*E*. *coli*), have been identified as significant causes of poultry mortality. Poultry can serve as potential sources of *E*. *coli* transmission, even when asymptomatic, posing a substantial threat to food safety and human health. The in ovo administration of antimicrobials is crucial for preventing and/or effectively combating acute and chronic infections caused by poultry pathogens. To achieve this goal, it is critical that antimicrobials are properly injected into embryonic fluids, such as the amnion, to reach target tissues and trigger robust antimicrobial responses. Several protocols based on antimicrobials were evaluated to meet these requirements. This review analyzed the impacts of antimicrobial substances injected in ovo on the control of *E*. *coli* in poultry. The reduction in infection rates, resulting from the implementation of in ovo antimicrobials, combined with efforts aimed at hygienic-sanitary action plans in poultry sheds, reinforces confidence that *E*. *coli* can be contained before causing large scale damage. For example, antimicrobial peptides and probiotics have shown potential to provide protection to poultry against infections caused by *E. coli*. Issues related to the toxicity and bacterial resistance of many synthetic chemical compounds represent challenges that need to be overcome before the commercial application of in ovo injection protocols focused on microbiological control.

## 1. Introduction

The establishment of microbiota in the eggshell may or may not influence healthy embryonic growth. It has been suggested that embryos may be resistant to bacterial infections originating in the eggshell, thanks to transgenerational immunological benefits [1]. On the other hand, it has been reported that the frequency of dead chick embryos with neck and beak deformities during the late incubation period may be associated with the *Escherichia coli* (*E*. *coli*) infectious process [2]. This microorganism is commonly found on eggshells [3]. The colonization of the microbiota in the eggshell begins in the hen’s oviduct [4], raising questions about the possible negative effect of oviductal bacteria on embryonic development. However, microbiota colonization of freshly laid eggshells has received greater attention given the recognized association with embryonic infections resulting from bacterial penetration [5].

Research has revealed several active agents, mainly with antibacterial effects, for the treatment of hatching eggshells after collection [6,7,8,9,10,11,12,13,14,15]. Oliveira et al. [10] reported that one hour after spraying a 0.39% clove essential oil solution on hatching eggs, the count of total aerobic mesophilic bacteria (−1.19 log) and Enterobacteriaceae (−1.19 log) in eggshells significantly reduced. Cantu et al. [9] demonstrated that spraying 3% hydrogen peroxide followed by immediate exposure to UVC light (254 nm) significantly reduced aerobic plate counts (−3.51 log) on the surface of the hatching eggshell. However, before applying sanitizers, it is crucial to consider that a specific microbial load may have penetrated or already have been present in the internal contents during egg formation in the oviduct. Direct treatment of the egg contents may be beneficial, as it is not yet clear what proportion of sanitizer residues applied to eggshells have to penetrate and perform their antimicrobial role internally. Given that the poultry embryo is the most important figure in poultry production, it is essential to guarantee their development away from any microbiological risk that would make their survival unfeasible at any stage. Therefore, it is hypothesized that the injection of antimicrobial substances directly into the internal contents of the egg during embryonic development represents a strategy to ensure more effective protection of embryos against microbial action, thus seeking to protect them from possible infections after hatching.

To address this issue, this review analyzed the impacts of antimicrobial substances injected in ovo on controlling *E*. *coli* infections in poultry.

## 2. Consultation of Published Studies

This review was prepared based on bibliographical research, consulting studies indexed on Google Scholar. The terms used were “in ovo injection”, “antimicrobial substances in ovo”, “eggshell”, “eggshell contamination”, “microorganisms in eggshells”, “eggshell penetration”, “eggshell antimicrobial defense”, “albumen”, “albumen antimicrobial defense”, “yolk”, “yolk antimicrobial defense”, “poultry embryonic infection”, “ *E*. *coli* in hatching eggs”, “*E*. *coli* in poultry embryos”, “poultry infected by *E*. *coli*”, “in ovo antimicrobials to control *E*. *coli*”, and “humans infected with *E*. *coli*”. The criteria adopted for inclusion included: original articles and reviews written in English or Portuguese; studies that investigated the eggshell; studies focused on administering antimicrobial substances through the in ovo technique; and studies related to microbial contamination of eggs and embryos, specifically with *E*. *coli*. Any studies that did not meet these inclusion criteria were promptly excluded from the analysis. The literature was consulted until the writing of each topic was finalized.

## 3. Eggshells and Their Natural Defenses

**Figure 1:**
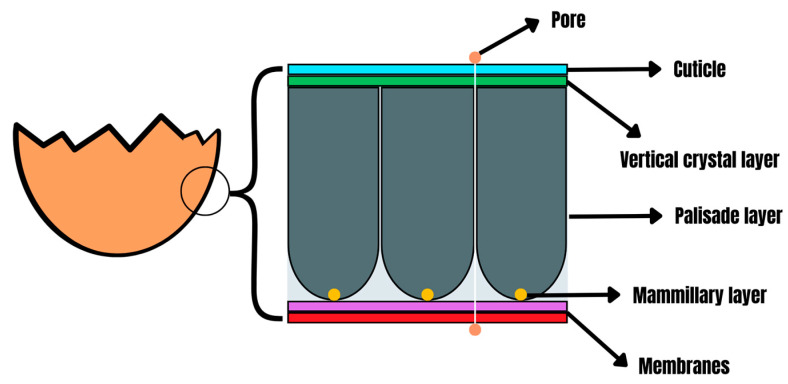
Poultry eggshell structure.

The eggshell generally has two predominant functions: nourishing and protecting the embryo. This protective function encompasses defense against pathogens, which is effective thanks to the interaction between the physical barrier capacity and the antimicrobial proteins present in the eggshell [16]. In addition to having pores, the eggshell is subdivided into the cuticular, vertical crystal, palisade, and mammillary layers, and the outer and inner membranes (Figure 1) [17]. The cuticle is the upper layer, rich in polysaccharides, hydroxyapatite crystals, lipids, and glycoproteins [18]. The eggshell comprises the lower layer, the vertical crystal, formed by crystals aligned perpendicular to the surface, the palisade layer, composed of calcite crystals embedded in an organic matrix, and the mammillary layer, consisting of calcified columns and cones that penetrate the shell membranes [19]. The inner layers, formed by the outer and inner membranes, represent the basal protective layer of the eggshell, composed of protein fibers [17,19].

Over the years, several studies have explored different possibilities as to how pathogens can overcome eggshell barriers (Table 1). The channels that influence the penetration of microorganisms into the eggshell can be significantly linked to poultry, egg, microorganisms, or environmental conditions.

## 4. *Escherichia coli* (*E*. *coli*) as a Threat during and after Embryonic Development

*E*. *coli* is a harmful pathogen in avian infections. This Gram-negative bacterium belongs to the Enterobacteriaceae family and can thrive in both aerobic and anaerobic environments, demonstrating adaptability when growing at temperatures ranging from 18–44 °C [30]. It may represent the most predominant bacteria among those isolated from eggs, shell-dead embryos, and newborn chicks [31]. *E*. *coli* can progress from harmless and asymptomatic colonization of the eggshell to the onset of potentially fatal embryonic diseases [32]. Its pathogenic specificity becomes particularly evident in embryonic infections, where *E*. *coli* demonstrates a remarkable ability to colonize the eggshell, invade it, and colonize embryonic tissues [33]. The invasion of *E*. *coli* through the eggshell not only represents a direct threat, but also promotes the invasion of other bacteria, such as *Staphylococcus aureus*, which is associated with high rates of embryonic mortality [34]. Among the main complications resulting from embryonic *E*. *coli* infection that lead to embryonic death are septicemia, omphalitis, and congenital deformities [2,35,36]. The presence of *E*. *coli* can result in the death of up to 92% of affected embryos [32]. Wang et al. [33] revealed that chick embryos died 48 h after being infected by *E*. *coli*. These findings provide an explanation for the decreased hatchability rate of *E*. *coli*-infected embryos at 18 days of development [37].

Another worrying aspect is the possibility of infection of embryos by *E. coli* through the eggshell, without them showing clinical signs during development. Such symptoms can appear after hatching [38], substantially increasing the risk of cross-contamination outbreaks and widespread mortality in poultry houses. Undesirable effects have been identified in broiler chickens infected by *E*. *coli*, manifesting through clinical signs and histopathological lesions such as: (1) Ruffled feathers, (2) inappetence, (3) respiratory manifestations, (4) sitting on hocks, (5) yellow and whitish diarrhea, (6) pericarditis, (7) enteritis, (8) airsacculitis, (9) liver and lung congestion, and (10) myocardial degeneration [39].

## 5. *Escherichia coli* (*E*. *coli*) as a Threat to Human Health

**Figure 2:**
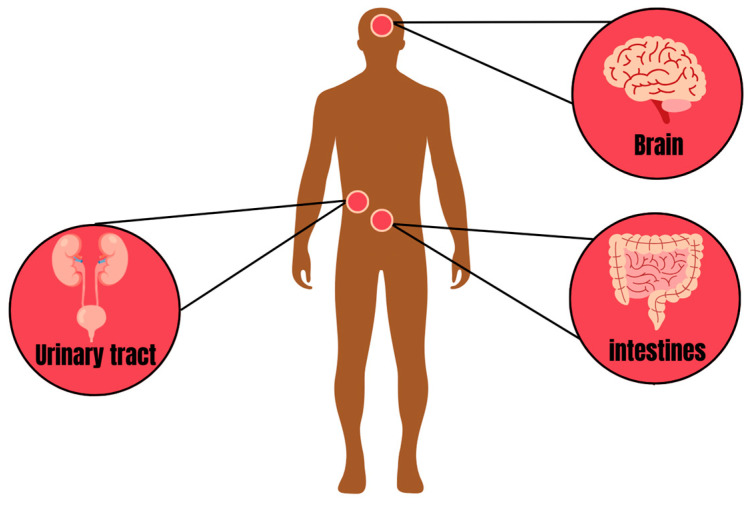
Common target sites of *E. coli* infection in humans.

Although this review does not directly focus on human health, it is imperative to recognize that the seriousness of microbial contamination in hatcheries, poultry farms and slaughterhouses cannot, under any circumstances, be ignored. Human health must always prevail over any poultry production process. Both ingestion and inhalation are crucial routes of direct exposure to microbial contamination in humans, covering both occupational and non-occupational contexts. Hatcheries, poultry farms and slaughterhouses pose potential risks to humans, both in terms of contamination through inhalation and the possibility of ingestion if the final products intended for consumption are contaminated, as these products are considered one of the main reservoirs of *E*. *coli* [40]. An additional concern arises when products initially supplied to commercial establishments, in accordance with microbiological standards, end up suffering contamination during storage, especially if this occurs under inadequate climatic and sanitary conditions. The consequences resulting from the inhalation or ingestion of *E*. *coli* can manifest themselves in humans as acute or chronic infections, compromising the integrity of human health. Some such infections include urinary infections that may or may not be associated with cases of bacteremia [41], intestinal problems, including diarrhea [42], and meningitis, associated with significant mortality rates, or with a high risk of developing serious neurological sequelae [42,43] (Figure 2).

## 6. What Is the In Ovo Injection Technique?

Antimicrobials administered into the egg via injections may represent an effective and rapid regimen to ensure microbial suppression during embryonic development and post-hatch. This regime, known as “in ovo injection”, aims to deposit a compound of interest in the internal environment of the egg via the intervention of qualified professionals [44]. Approximately forty years ago, researchers tested this regime in the laboratory for vaccinating poultry before hatching [45]. Today, its commercial application around the world continues to prioritize vaccination as its main objective. Based on research already carried out [46,47,48,49,50,51], in ovo injections offer a range of advantages in poultry farming, such as:

- The in ovo injection technique does not require very complex professional training to be administered.
- It can be considered the best option for early and systemic immunization of poultry, with the absence of pain and stress.
- This technique allows for rapid and effective absorption of the injected medication, leading to faster immunization, or a faster response to treatment.
- It can inhibit bacterial growth and multiplication, thus reducing the cross-spread of bacteria in hatcheries and farms, as well as outbreaks of fatal diseases.
- It can induce long-lasting immunity, ensuring that poultry protection is maintained over time.
- It can favor the achievement of productivity gains related to the effects of the injected compound.
- It precipitates a reduction in operational and treatment costs related to poultry farming.

**Figure 3:**
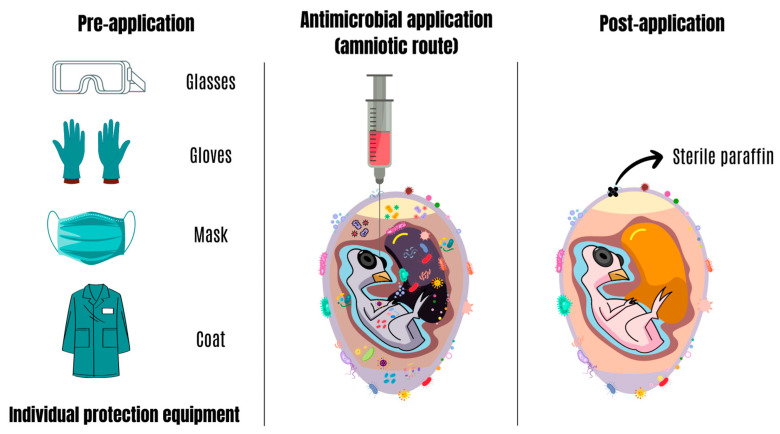
Antimicrobial intervention through in ovo application. Source: Adapted from Oliveira et al. [7].

Firstly, before carrying out the in ovo injection technique, individual safety equipment must be used (Figure 3). The use of syringes with sterile needles and appropriate calibers for eggs is crucial, as it must be a minimally invasive and painless protocol. In general, the amount of substance injected is 0.1 mL [52], although a larger volume may be considered [53]. However, it is essential to highlight that, depending on the nature of the substance, the injection volume cannot exceed 0.4 mL, as this practice may be related to undesirable productive effects [54]. Additionally, 1 mL syringes with 23-G and 1-inch needles have been efficiently used in this practice [55]. After application, sterile paraffin is normally used to seal the pierced egg [56]. Although the recommendations above are not a general rule, the absence of adequate conditions, specifically for each antimicrobial substance, can significantly increase the risk of failures and embryonic mortality in the in ovo injection process [54]. The anatomical region of the egg used to administer antimicrobials is relevant to the safety and effectiveness of treatments developed to prevent or treat avian microbiological complications until post-hatch. Thus, the amnion, an extra-embryonic membrane, has been recommended as a potential site for the direct delivery of antimicrobial substances [48] (Figure 3). This intervention can occur during the prenatal or perinatal phases of embryonic development [51]. After the intervention, an immediate and prolonged microbial reduction is expected.

**Figure 4:**
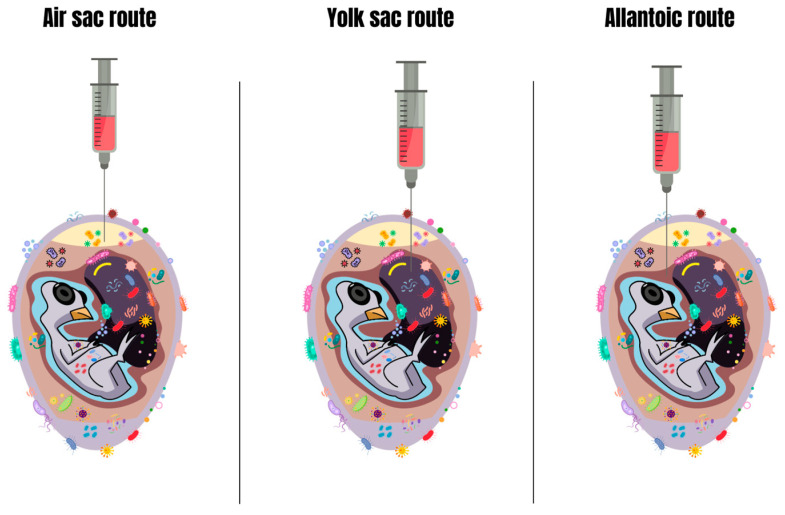
Other routes of drug application in ovo. Source: Adapted from Oliveira et al. [7]

To understand the in ovo drug administration route, it is necessary to understand the physiology of embryonic development. Among the various in ovo injection routes, the amniotic route, as mentioned earlier, is the most popular approach for in ovo drug administration. The main advantage of the drug administration system via the amniotic route, compared to other in ovo delivery routes (Figure 4), is the rapid distribution of the compound to the embryo. According to Williams [47], after being deposited in the amnion, therapeutic substances are rapidly absorbed orally and through the mucosal surfaces of the embryo’s respiratory and digestive tracts. Antibacterial therapies require that pharmacological agents act quickly on the body of the target organism to provide protection and/or treatment. In this context, the amnion stands out as the best option for prevention or treatment against *E*. *coli* in embryos, since the drugs deposited in it normally have an efficacy rate above 90% [57].

## 7. In Ovo Injection as a Front Line against *Escherichia coli* (*E*. *coli*) Infection in Poultry

The management of infectious diseases in poultry requires daily administration of antimicrobials for a period that varies according to the target bacteria and its susceptibility to the antimicrobial, the severity of the infection, the immunological status of the poultry, and administration standards defined by the manufacturer, among others. However, non-adherence to therapy by poultry can lead to recurrence of the disease. Therefore, it is more advantageous to adopt preventive management practices even before signs of avian infection appear. The use of injectable antimicrobial formulations in the egg during embryonic development emerges as an effective preventive practice against microbial infection in poultry, especially by *E*. *coli* (Table 2). However, it is worth highlighting the importance of being cautious when using antibacterials for this purpose, mainly due to the development of antibacterial resistance. It is hypothesized that this efficiency of the in ovo injection practice is due to the rapid distribution of the antimicrobial throughout the body and its prolonged action. Twenty-four hours after administration into the amnion, the antimicrobial substance may have already spread throughout the embryo’s body, including the gastrointestinal tract, respiratory system, and skin [48]. The effectiveness of the antimicrobial administered in ovo can allow the survival of 90% of embryos against *E*. *coli* infection [58] and ensure the protection of 100% of chicks against yolk sac infection by the same microorganism [59]. Furthermore, the antimicrobial effect of the injectable substance in the egg can be observed in poultry even when they reach 21 days of age [49].

**Figure 5:**
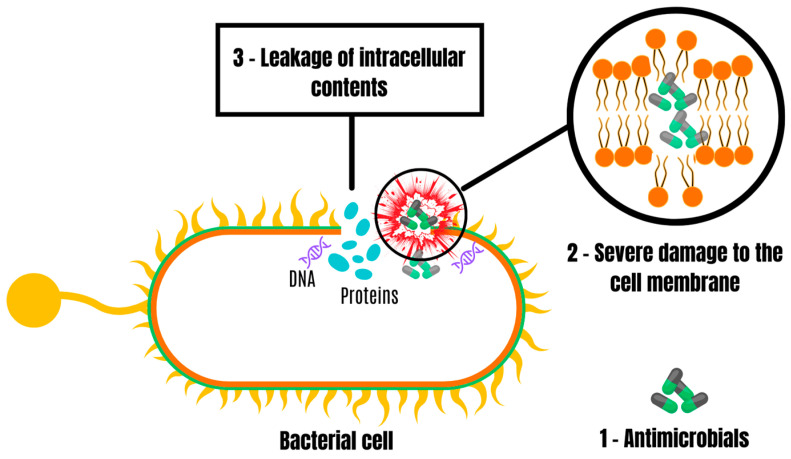
One of the mechanisms of action of antibiotics on bacteria.

As noted previously, successful antimicrobials demonstrate high efficacy in long-lasting prevention and rapid treatment of poultry infection-causing pathogens such as *E*. *coli*, providing systematic protection that effectively limits or prevents the spread of infection in farming systems. Many antimicrobials demonstrate success in combating *E*. *coli* due to their action mechanisms that result in the death of this bacterium. It has been elucidated that natural antimicrobials may have the ability to cause damage to the cell membrane of *E*. *coli*, resulting in the leakage of proteins and nucleic acids (Figure 5). This phenomenon triggers the destabilization of metabolic activity, ultimately culminating in bacterial cell death [70]. In the same way, synthetic chemical antimicrobials can also induce disturbances in the cell walls and membranes of *E*. *coli*, reducing its protection and resulting in the loss of intracellular content [71]. This is the most elucidated antibacterial mechanism.

Advances in preventing or treating *E*. *coli* infections through in ovo delivery of substances have primarily focused on the use of antimicrobial peptides and probiotics. A peptide is a chain of amino acids that generally does not exceed 50 amino acids, linked together by peptide bonds [72]. Identified sources of peptides include microorganisms, plants, animals, and humans [73]. Peptides present a cocktail of attractive characteristics, such as compatibility with poultry safety [65]. Furthermore, they have pharmacological aspects, including activity against gram-positive and gram-negative bacteria [65,74]. The implementation of antibacterial peptides in poultry farming can significantly contribute to solving several problems related to poultry productivity and health [75]. Two important families of antimicrobial peptides with potential application in poultry farming are β-defensins and cathelicidins [76].

Probiotics are beneficial live microorganisms that, in certain concentrations, exert a broad spectrum of biological activities. This includes antibacterial properties, which have played a significant role in increasing interest in opening new therapeutic horizons in poultry farming [77,78]. A review carried out by Cox and Dalloul [79] on the role of probiotics in poultry concluded that probiotics are beneficial for improving performance, maintaining healthy balance of the intestinal microbiota, and neutralizing adverse effects of infectious diseases. Several microorganisms have physiological and technological characteristics that classify them as probiotics. Among them are *Lactobacillus acidophilus*, *Lactobacillus casei*, *Lactobacillus fermentum*, *Lactobacillus gasseri*, *Lactobacillus johnsonii*, *Lactobacillus lactis*, *Lactobacillus paracasei, Lactobacillus plantarum, Lactobacillus reuteri, Lactobacillus rhamnosus*, *Lactobacillus salivarius*, *Bifidobacterium longum*, *Bifidobacterium bifidum*, *Bifidobacterium breve*, *Bifidobacterium animalis*, and *Streptococcus thermophilus* [80].

In short, it has been observed that the antibacterial compounds injected into the egg act mainly to reduce the bacterial load of the poultry, protecting them against infections before or after hatching. Furthermore, they beneficially modulate the intestinal microbiota and strengthen the poultry immune response, minimizing cases of mortality.

## 8. Is In Ovo Injection Harmful to Hatchability?

Hatchability is the gold parameter for evaluating antimicrobial techniques involving embryos and hatchery performance. It represents the proportion of chicks born alive for a specific sample of eggs [51]. A recent bibliographic mapping addressing the relationship between hatchability and the in ovo injection technique [51] showed that, in general, the practice of in ovo injection tends to improve hatchability. However, the review highlighted that this technique has a more significant impact on poultry health parameters than on hatchability itself, and that association of the technique with possible loss of hatchability was observed in specific cases [51]. Therefore, it is more interesting for the poultry sector to adopt sanitary procedures, with the potential not only to ensure poultry is free from bacterial infections but also to at least preserve hatchability, given that poultry yields depend significantly on this index and high-quality standards of the poultry. Choosing a multifunctional antimicrobial can also minimize costs that could otherwise make the adoption of in ovo infection unfeasible. A wide repertoire of antimicrobial solutions, such as carbohydrate/electrolyte + potassium chloride + theophylline, tripotassium citrate + potassium chloride + theophylline, creatine + potassium chloride + theophylline [81], the nano form of zinc, copper, or selenium [82], vitamin A, vitamin E, vitamin D3, folic acid, [83] L-Arginine, and L-Threonine [84], were not associated with harm in hatchability.

## 9. Antimicrobials and Hygiene Practices in the Poultry Sector

Eggshell contamination by *E*. *coli* often originates in breeding sheds. Subsequently, this contamination can be transmitted horizontally to the embryo, persisting until after hatching. Furthermore, poultry can be directly contaminated by *E*. *coli* present in the shed environment. Therefore, poultry houses with unsanitary and microbiologically compromised conditions can negatively affect the quality of poultry and act as sources of inoculum for pathogenic microorganisms, such as *E*. *coli*, which can cause significant damage to poultry production and the safety of poultry food products. These unsanitary conditions also have the potential to obstruct trade in poultry products in both national and international markets. To prevent infectious outbreaks caused by *E*. *coli* and poultry health emergencies at regional, national, or international levels, it is essential to implement preventive microbial control programs. This includes effective safety management before, during and after production. The use of antimicrobials selected based on antibiograms, under the guidance of qualified professionals and in partnership with poultry companies, is a key component of these programs, ensuring effective disease prevention.

Ahmed et al. [85] showed that the application of 250 mL of chlorine dioxide (ClO_2_) for fumigation in a broiler shed at the end of 5 weeks of rearing resulted in a significant reduction in the concentration of *E*. *coli* in just 10 min. This reduction remained significantly effective up to 12 h after application, without causing any adverse effects to the health of the poultry. Likewise, Jiang et al. [86] presented results indicating that spraying a poultry house with a sanitizer containing aldehydes, quaternary ammonium salt, and alcohol (ratio 1:1500) resulted in a significant reduction in the relative abundance of pathogens of the genus *Escherichia*-*Shigella*. Based on these studies, the importance of a detailed management plan that incorporates antimicrobial actions in poultry sheds is reinforced. However, the efficiency of the plan depends on the daily execution of these actions, as well as the training of the professionals responsible for their execution [13].

## 10. Conclusions and Future Perspectives

The in ovo injection technique allows the development of personalized protocols to overcome specific challenges in the effective administration of antimicrobials and combating *E*. *coli* infections. The integration of this technique with practices already established in the poultry sector, such as rigorous hygienic-sanitary maintenance in sheds, can enhance the efficiency and precision of treatment, ensuring a more targeted and effective approach to poultry care. Although this technology has great potential, it is crucial to address some issues before its full implementation in industrial poultry environments. For example, additional research is essential to evaluate the effectiveness of combining eggshell sanitation and in ovo antimicrobial administration, specifically to improve the practice of in ovo injection in poultry production, especially in the treatment of *E*. *coli*. Furthermore, before proceeding with its large-scale adoption in poultry farming, it is vital that the combined technology undergoes rigorous testing to ensure its safety for poultry, humans, and the environment. It is hoped that this review will provide poultry researchers and professionals with a clear perspective on how careful selection of antimicrobials, combined with a refined in ovo application protocol, can constitute an effective strategy to significantly optimize yields in the poultry sector.

In the practice of in ovo injection in the poultry industry, it is expected that, in addition to vaccination, there will be a routine dedication to the in ovo delivery of antimicrobials, with the main objective of controlling bacterial proliferation. However, this requires a careful and comprehensive approach to several issues, such as:

- Over the past few decades, several protocols have been developed for the delivery of substances in ovo in the poultry field. Some of these protocols have been specifically designed to protect poultry against bacterial infections. Within these protocols, the use of antimicrobial peptides and probiotics has been the subject of intense investigation and reporting. The implementation of these protocols, centered on such compounds, takes priority in commercial production, given the concentrated database available that supports their characteristics of simplicity, cost-benefit, ease of in ovo application, and compatibility with poultry safety. In addition, the toxicity and bacterial resistance of many synthetic chemical compounds have been considered.
- The chosen in ovo delivery route may influence the effectiveness of antibacterials for poultry. Therefore, studies have proposed the amniotic route as the most effective to guarantee avian protection. These results will contribute to the development of commercial protocols utilizing a more advantageous in ovo delivery route.
- Some tested compounds may exhibit antibacterial specificity for a specific group of bacteria, meaning that the compound does not have a broad antibacterial spectrum. Although this review focuses on the control of *E*. *coli*, the search for compounds with broad-spectrum antibacterial properties represents a promising avenue for in ovo injection protocols. This requires further investigation.
- When developing in ovo application protocols, it is crucial to consider the associated economic cost and environmental damage. High costs can create barriers to commercial application, while the use of toxic synthetic chemicals can pose a threat to the environment.
- Many of the compounds tested in ovo were only evaluated under laboratory conditions. Therefore, testing under commercial conditions is essential, since the results obtained in the laboratory may encounter several limitations, even if minimal, due to the different realities faced in practice.

## Figures and Tables

**Table 1:** Some factors associated with microbial penetration into the eggshell.

Factors	Reference
Absence or partial deposition of the cuticle	[20]
Eggshell pore diameter	[20]
Exposure of the egg to temperature variation regimes	[21]
Translucent eggshell surface	[21]
Genetic origin of Poultry	[22]
Egg dynamic stiffness	[23]
High contamination of the eggshell surface	[23]
Motile and non-clustering properties of some microorganisms	[24]
Poultry housing system	[25]
Poultry feed	[25]
Washing and sanitizing methods	[26]
Egg storage time	[27]
Number of pores in the eggshell	[27]
Eggshell condensation	[28]
Newly laid eggs (immature cuticle)	[29]
Chemical composition of the cuticle	[29]

**Table 2:** Control of *E*. *coli* in poultry after application of antimicrobials in ovo.

Compound Classification	Concentration	Day of Application in Embryos	Application Location	Effects Found after Application	Study
**Immune stimulants**					
Cytosine-phosphodiester-guanine oligodeoxynucleotides + polyphosphazene	50 µg/100 µL	E18	Amnion	Increased the immunoprotective effect against *E*. *coli* infections in poultry	[46]
Cytosine -phosphodiester-guanine oligodeoxynucleotides	50 µg/100 µL	E18	Amnion	It can be used to prevent and control mortality due to yolk sac infection by *E*. *coli*	[60]
**Probiotics**					
Intestinal microbial product	3.3 × 10^5^ viable bacteria/egg	E18	Amnion	Reduced the abundance of Enterobacteriaceae (a family that includes *E*. *coli*) in the intestinal microbiota	[61]
*Bacillus* spp. probiotic-based	5 × 10^7^ CFU/mL (1 × 10^7^ CFU/200 µL)	E18	Amnion	Reduced the severity of virulent horizontal transmission of *E*. *coli* and infection of poultry in the incubation cabinet	[62]
Lactic acid microbiota	10^7^ CFU/mL	E19	Amnion	Reduced Enterobacteriaceae colonization in poultry after *E*. *coli* infection	[63]
*Bacillus subtilis*, *Pediococcus acidilactici*, and *Enterococcus faecium*	10^7^ CFU/mL	E18	Amnion	Reduced the intestinal population of *E*. *coli* in poultry	[64]
**Antimicrobial peptides**					
Avian antimicrobial peptides	30 µg peptide/100 µL PBS/embryo	E18	Amnion	Effective protection against yolk sac infection caused by *E*. *coli*	[65]
Chicken cathelicidin analog DCATH-2	4.4 mg/mL/100 µL PBS/embryo	E18	Amnion	Protected poultry against *E*. *coli* infection	[48]
**Prebiotics**					
Raffinose and stachyose	5 and 10%	E17	Amnion	The concentration of *E*. *coli* in the intestinal content of poultry did not show significant variations	[66]
**Nanomaterials**					
Green Silver Nanoparticles	0.17 mg/mL	E17.5	Amnion	Reduced *E*. *coli* counts in the cecal content of poultry	[67]
**Bacteriophages**					
Phage cocktail	100 µL of the phage cocktail (5.2 × 10^8^ PFU/mL) or DPBS	E16	Allantois	Prevented the development of avian colibacillosis	[50]
**Synbiotics**					
*Lactobacillus plantarum* + Astragalus polysaccharide	200 µL of the *Lactobacillus plantarum* + 2 mg/egg Astragalus polysaccharide	E18.5	Amnion	Increased colonization of *Lactobacillus* spp. and *Bifidobacterium* spp. and decreased the population of *E*. *coli* in the avian cecum.	[49]
**Natural extract and vitamins**					
Grape seed extract and vitamin C	Grape seed extract (3, 4.5 or 6 mg/egg), and vitamin C (3 mg/egg)	E18	Air sac	Decrease in the population of *E*. *coli* in the ileum	[68]
**Amino acids**					
L-arginine	1–0.5%	E14	Amnion	Reduced *E*. *coli* in the cecum of poultry	[69]

## Data Availability

Not applicable.
